# Supplementary material for: Repair of oxidized methionine residues in the chaperone Spy maintains periplasmic proteostasis under chlorite stress in Escherichia coli
Source: PLoS Biol. 2025 Sep 29;23(9):e3003411. doi: 10.1371/journal.pbio.3003411 (PMC12503329; doi:10.1371/journal.pbio.3003411)
Supplement: S1 Raw Images — The imaging method used to capture the blots is indicated. Lanes marked with an ‘X’ correspond to those not included in the final figures. (PDF) [file pbio.3003411.s009.pdf]

α-Spy

Chemiluminescence signal was collected using an ImageQuant Las4000 camera (GE Healthcare).

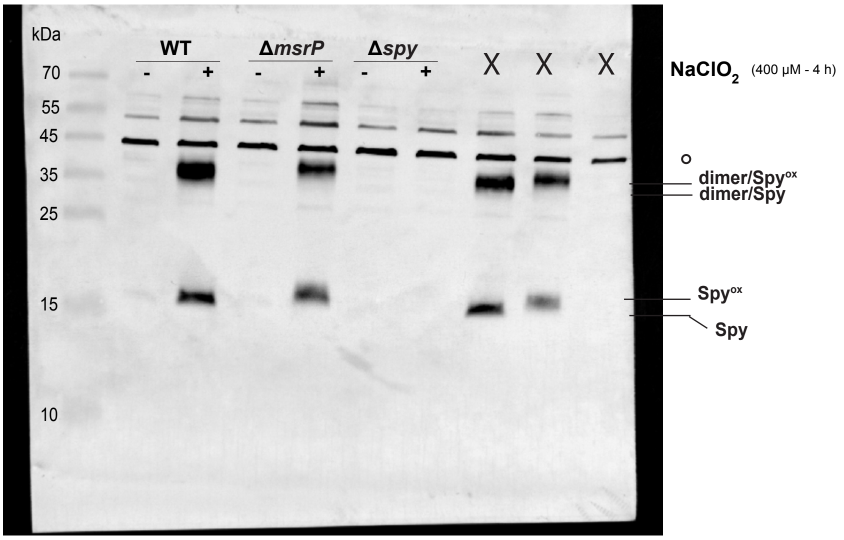

Raw image
Original images for blot Fig 1B

α-Spy Chemiluminescence signal was collected using an ImageQuant Las4000 camera (GE Healthcare).

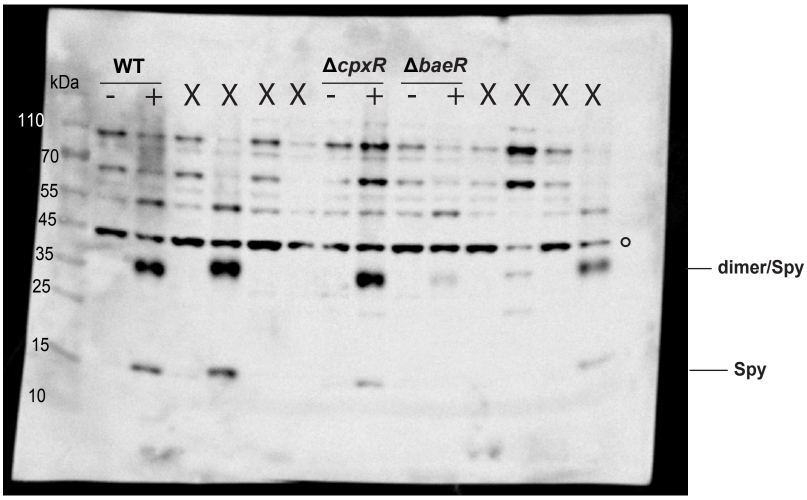

Raw image
Original images for blot Fig 1C

α-Spy

Chemiluminescence signal was collected using an ImageQuant Las4000 camera (GE Healthcare).

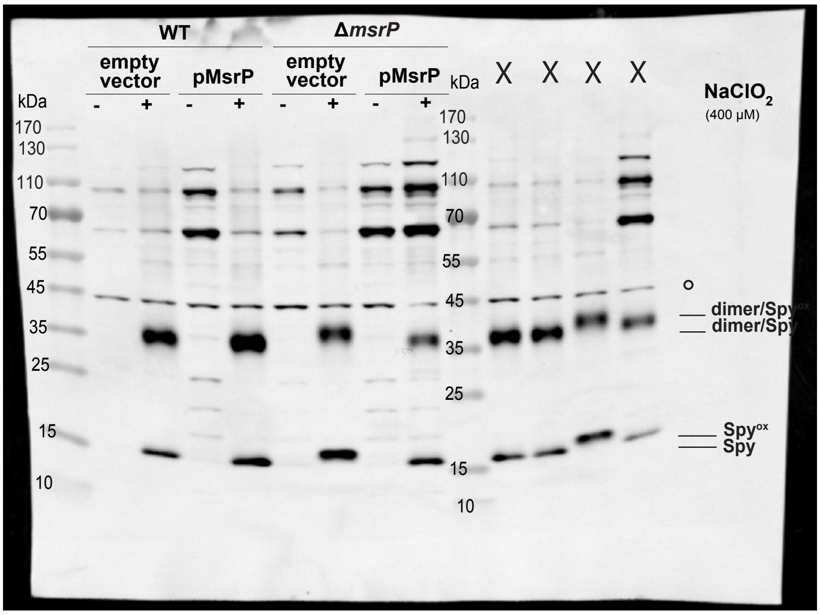

α-MsrP

Chemiluminescence signal was collected using an ImageQuant Las4000 camera (GE Healthcare).

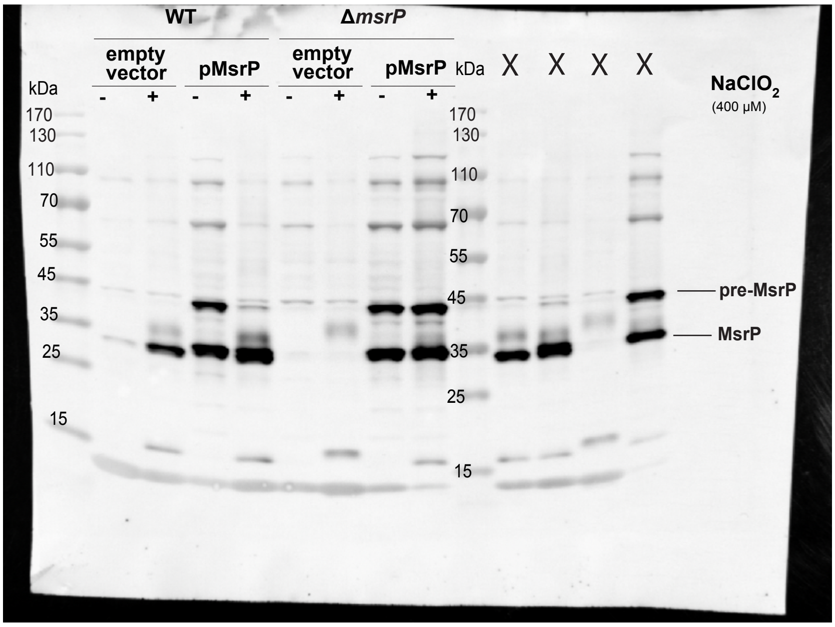

The same membrane was first probed with an anti-Spy antibody, and subsequently reprobed with an anti-MsrP antibody.

Raw image
Original images for blot Fig 1D

Chemiluminescence signal was collected using an ImageQuant Las4000 camera (GE Healthcare).

α-Spy

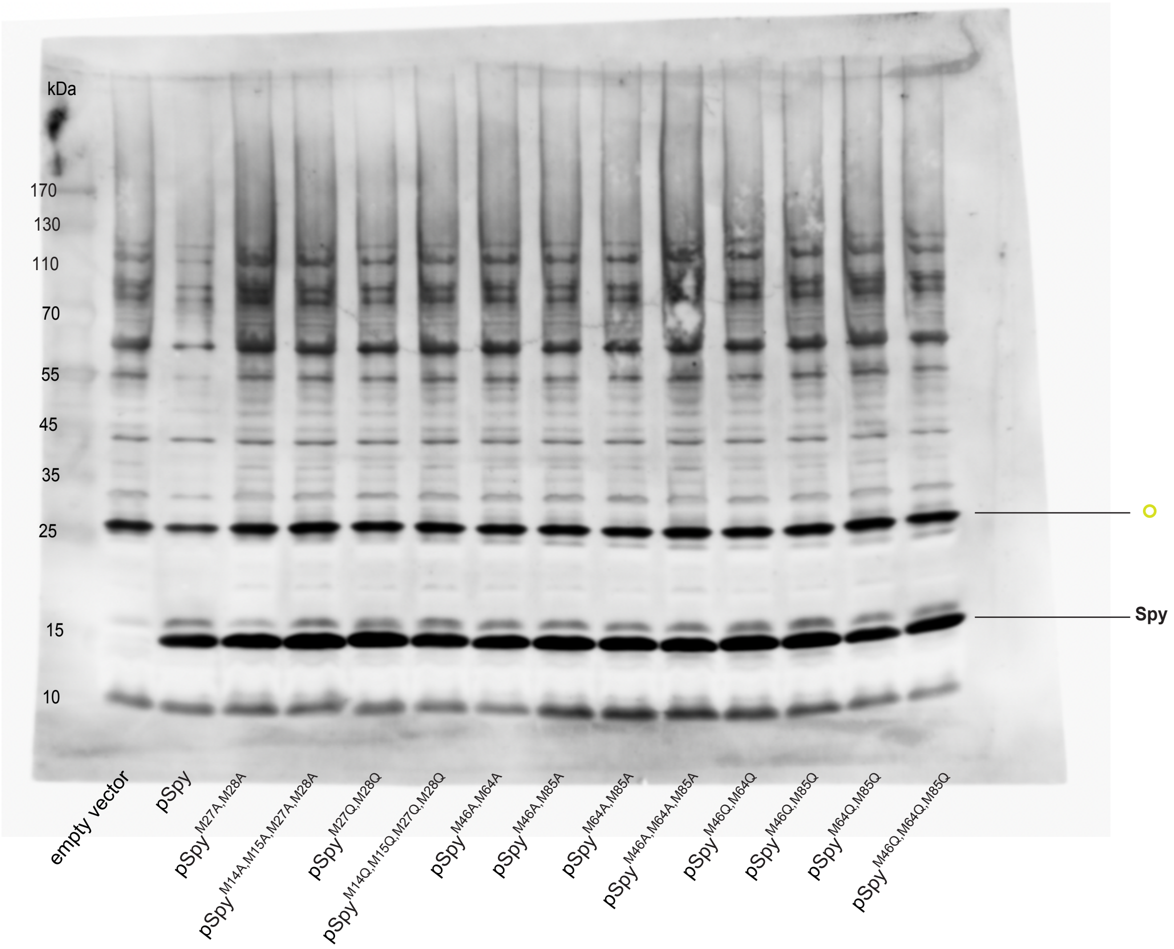

Raw image
Original images for blot Fig 3D

α-Spy

Chemiluminescence signal was collected using an ImageQuant Las4000 camera (GE Healthcare).

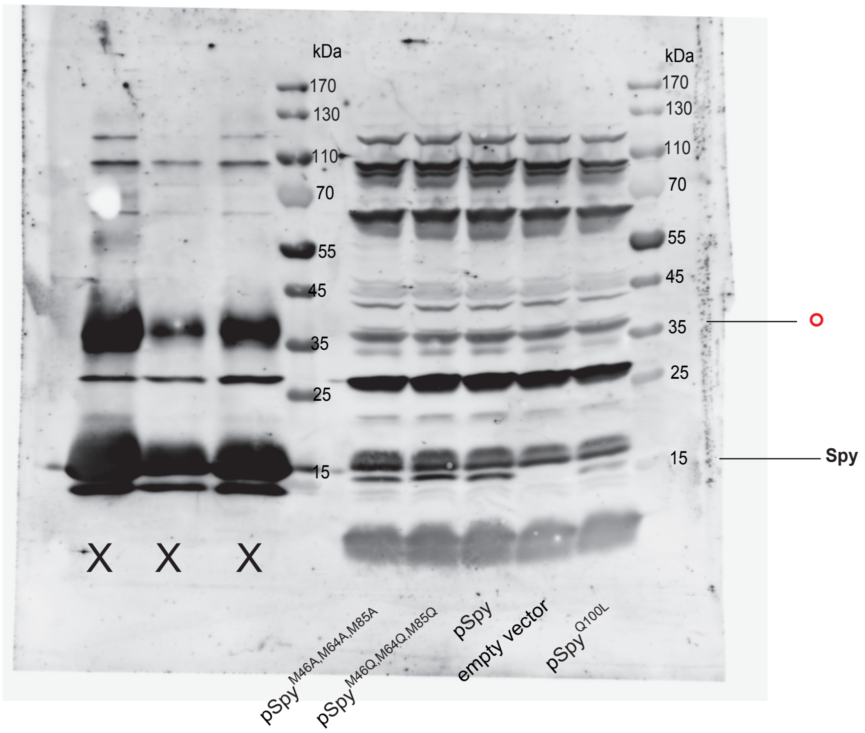

Raw image

Original images for blot Fig 4B

α-Spy

Chemiluminescence signal was collected using an ImageQuant Las4000 camera (GE Healthcare).

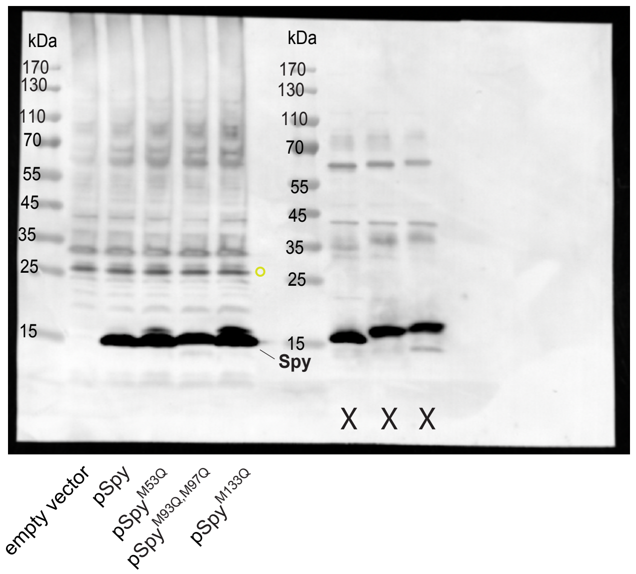

Raw image
Original images for blot S3 Fig
