# Supplementary material for: Repair of oxidized methionine residues in the chaperone Spy maintains periplasmic proteostasis under chlorite stress in Escherichia coli
Source: PLoS Biol. 2025 Sep 29;23(9):e3003411. doi: 10.1371/journal.pbio.3003411 (PMC12503329; doi:10.1371/journal.pbio.3003411)
Supplement: Attachment — Submitted filename: reponses reviewers.pdf [file pbio.3003411.s012.pdf]

Aix Marseille Université — LABORATOIRE DE CHIMIE BACTÉRIENNE — cnrs

Responses to editor and reviewers (comments in red / responses in black), modifications in the text are highlighted in yellow.

Referee #1

The work by Loiseau et.al presented an interesting discovery that the periplasmic chaperone Spy is susceptible to chlorite-induced oxidative damage, and the MsrPQ system is responsible to maintaining Spy's activity by reducing its oxidized methionine residues modified under chlorite stress. Overall, the findings are novel, the experiments were well designed, and high-quantity data were obtained. However, I am not fully convinced with the conclusion that Spy is the key target of chlorite-induced oxidative damage and would suggest modifications to further strengthen the paper.

We thank the referee for their enthusiastic comments regarding the novelty, as well as the high quantity and quality of the experiments. We are especially grateful for their suggestions for complementary experiments, which have greatly strengthened our manuscript.

Major points:

1. I have no doubt that Spy's activity will be affected to some extent by methionine oxidation, but will question on the significance of this modification. The major conclusion were drawn from Spy activity assay where glutamine substitutions were used to mimic methionine sulfoxide. I have to admit that this is indeed a frequently used strategy, however, glutamine with additional amide group is different in polarity so it is possible that this substitution decreases Spy's activity mainly through perturbing the normal 3-dimensional folding of the protein, especially when these substituted methionine residues were within the hydrophobic patches on Spy's concave surface. In other words, I am afraid that the effect of methionine oxidation in Spy might be exaggerated with glutamine substitutions. Essential point: I would suggest the authors to perform the activity assay with Spy purified from the msrP-deletion strain treated with chlorite, or oxidize Spy in vitro using chlorite, and then assay its activity change.

The substitution of methionine residues with glutamine to mimic site-specific oxidation is indeed a widely used strategy. To our knowledge, this approach was first introduced by the Means and Squier groups to study the effects of methionine oxidation on calmodulin (Chin et al. 1996 and Bigelow et al. 2011). It was later employed with particular elegance by the Winter lab to demonstrate the activation of the transcription factor HypT of *E. coli* through methionine oxidation (Drazic et al. 2013). We have since made extensive use of this strategy, which has consistently proven to be highly effective (Henry et al. 2021, Vergnes et al. 2022, El Hajj et al. 2022).
However, the concern raised by the reviewer regarding this type of substitution is entirely valid. In this revised version, we first provide direct evidence of methionine oxidation occurring in vivo during chlorite stress. Following the reviewer's suggestion, we then performed chaperone activity assays using an oxidized form of Spy. For this, oxidation was carried out *in vitro* using H_2O_2, following a robust and well-established protocol. The oxidation state of Spy was confirmed by mass spectrometry. Our results show that the oxidized form of Spy loses its ability to prevent α-lactalbumin aggregation. As predicted by the reviewer, the loss of function of Spy-ox is indeed less pronounced compared to the Met→Gln variants. All of these results are presented in Figure 4 and described in lines 222-232.

C.N.R.S.- L.C.B. 31 Chemin Joseph Aiguier, 13402 Marseille CEDEX 20, France.
Tel. : (33) 4.91.16.40.77. Fax : (33) 4.91.71.89.14
Email : lcb-sec@imm.cnrs.fr

2. The authors demonstrate that the methionine residues on the disordered N-terminus were less critical to affect activity, while Met69, Met87, and Met108 are critical for Spy's chaperone activity. I wonder whether they investigated other conserved methionine residues also located on the concave surface of Spy, namely Met 76, 116, and 120. I bet substituting these residues also had some effect since they also localized in or near the hydrophobic patches. But even with this information, whether these residues were indeed the modification targets under chlorite stress is still in doubts. Is it possible that the authors provide direct evidence which methionine residue(s) in Spy are indeed oxidized upon chlorite treatment, for example, through mass spectrometry characterization?

Our initial strategy for selecting Spy methionine residues was based on hotspot, sequence conservation, and homology with CpxP. This approach can indeed be criticized for its lack of exhaustiveness. We thank the reviewer for pointing out the presence of additional methionine residues on the concave surface of Spy.
In this revised version, we substituted Met53 (76), Met93 (116), Met97 (120), and Met133 (156) with glutamine. The results indicate that positions 93 and 133 do not appear to be critical for Spy function. In contrast, the Spy(M93Q,M97Q) variant appears to be non-functional. Immunoblot analysis shows that the expression levels of these variants are comparable to that of Spy^{WT}. All of these results are presented in S2 Fig. and described in lines 184-190.

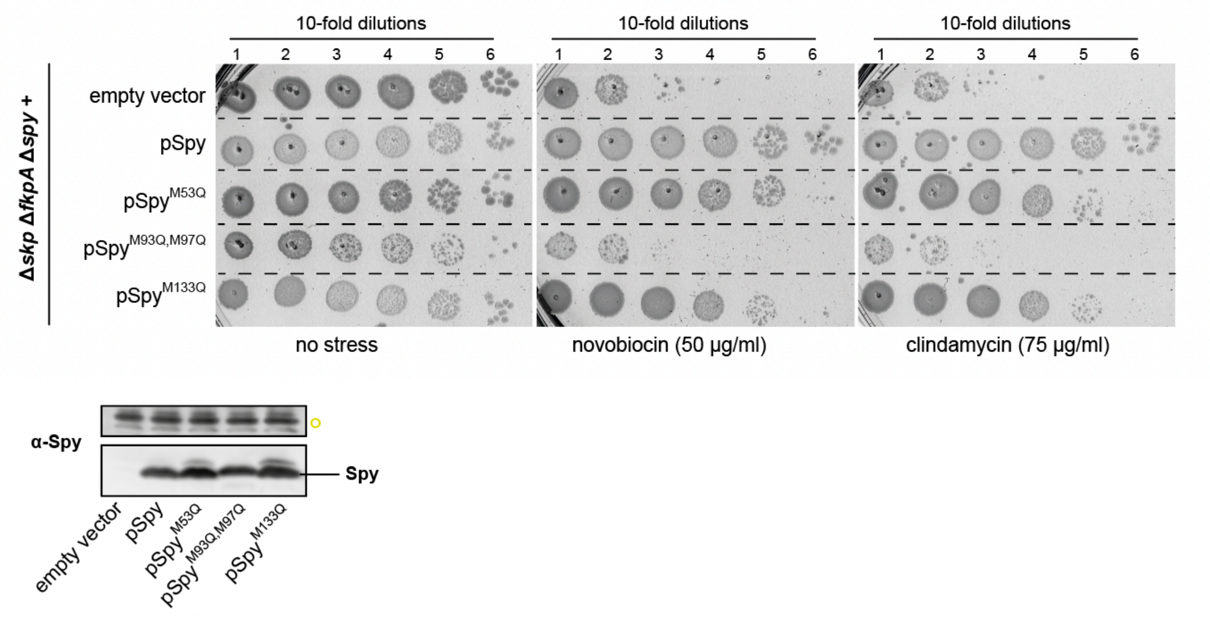

We thank the reviewer for this insightful comment highlighting the need for direct evidence of methionine oxidation in vivo during chlorite stress. To rigorously address this point, we devoted substantial effort to minimizing non-physiological artifacts associated with plasmid-based overexpression. Specifically, we constructed new strains carrying a chromosomally integrated Strep-tagged version of *spy*. We then analyzed methionine oxidation levels in Spy produced under native expression conditions—i.e., without plasmid overproduction—in both wild-type and Δ*msrP* strains subjected to chlorite stress. Our results demonstrate that methionine oxidation in Spy is significantly higher in the Δ*msrP* mutant compared to the wild-

C.N.R.S.- L.C.B. 31 Chemin Joseph Aiguier, 13402 Marseille CEDEX 20, France.
Tel. : (33) 4.91.16.40.77. Fax : (33) 4.91.71.89.14
Email : lcb-sec@imm.cnrs.fr

type strain. These findings are presented in Figure 1E and described in lines 139-145 of the revised manuscript.

3. The authors showed that MsrP is responsible to reverse the oxidation of Spy in vivo by the motility-shift assay in SDS-page, however, it is still unclear whether MsrP help to recover Spy's chaperone activity. Could the authors demonstrate whether the presence of absence of MsrP affect Spy's in vivo activity under chlorite stress, or with purified proteins, whether MsrP recovery the activity of chlorite-oxidized Spy?

We thank the reviewer for their comment regarding MsrP's ability to restore Spy activity. In agreement with PLOS Biology editors, we focused our efforts on demonstrating that, in vitro, the repair of oxidized Spy by MsrP effectively restores its chaperone activity. Our results show that the oxidized, non-functional form of Spy regains its ability to protect α-lactalbumin from aggregation upon treatment with MsrP.
Additionally, we performed mass spectrometry analysis to confirm the reduction of methionine sulfoxide residues in Spy by MsrP. All of these results are presented in Figure 4C-D and described in lines 222-232.

Minor points:
1. The numbering of the methionine residues was counted from the first residue including the signal sequence, but the Q100L is counted from the mature sequence of Spy. This disagreement should be solved.

This was an oversight on our part, and we thank the reviewer for pointing it out. The numbering has been thoroughly reviewed and corrected throughout the manuscript.

2. Molecular weights markers should be included in western blots.

The requested modifications have been made.

Reviewer #2 (Jan-Ulrik Dahl):

The bacterial periplasm is extremely vulnerable to a variety of environmental stressors, including oxidative stress caused by reactive oxygen and chlorine species (RO/CS). Previous work by the Ezraty crew nicely demonstrates that reduction of chlorate into chlorite induces the expression of MsrPQ, a periplasmic methionine sulfoxide reductase system that repairs oxidized methionine residues in periplasmic proteins. In this follow-up study, the authors now provide novel mechanistic details on the redox-regulated activity of the periplasmic chaperone Spy. Using proteomic and transcriptomic approaches, Loiseau et al. show that chlorite stress (induced either by chlorite addition or through chlorate reduction) elicits increased Spy expression, a response likely being regulated by the BaeSR TCS rather than by CpxR. The study further provides convincing evidence that Spy's oxidation, particularly of three methionine residues in its "cradle" region, impairs its chaperone activity and that MsrPQ helps restore Spy's functionality by reducing its oxidized methionine, ensuring periplasmic protein quality control.

The paper is straightforward and easy to read. All experiments were designed and conducted in a logical way with important controls included. I only have minor comments for the authors to consider:

Dear Dr. Dahl, we sincerely thank you for your extremely positive feedback on our study. We agree that uncovering this new mechanism under chlorite stress will significantly enhance our understanding of bacterial adaptation to RCS. We are very enthusiastic about continuing our research on this promising topic.

1) The visualization of the proteomic data in Fig 1A (and the supplementary data set) could be improved to make them more accessible to a broader audience. For instance, all significantly up- and downregulated proteins (i.e. >2-fold change) could be color-coded based on regulons and the protein name provided. Do the differentially expressed proteins fall into specific functional groups of interest, e.g. the BaeSR regulon?

We highlighted the most overproduced and underproduced proteins in red and blue, respectively. Additionally, we identified the top 10 proteins in each category (red and blue) to provide clearer and more direct information.

2) Given that Spy was the most upregulated protein in the proteomic study, can the authors comment on the oxidation state of three crucial Spy methionine residues?

This is a fair point, also raised by several reviewers. As addressed in response to Reviewer 1, Question 2, we performed mass spectrometry analyses on oxidized Spy protein, both in vitro (data shown in Figure 4) and in vivo, by comparing oxidation levels in a wild-type strain and an *msrP* mutant (data shown in Figure 1). The data provide valuable insight into methionine residues that are particularly sensitive to oxidation.

C.N.R.S.- L.C.B. 31 Chemin Joseph Aiguier, 13402 Marseille CEDEX 20, France.
Tel. : (33) 4.91.16.40.77. Fax : (33) 4.91.71.89.14
Email : lcb-sec@imm.cnrs.fr

3) Are the western blots representatives of several biological replicates and did those resulted in the same outcomes?

All experiments presented in this study were performed at least three times and consistently yielded similar results. We had omitted to specify this in the original figure legends. This has now been corrected in the revised version of the manuscript.

4) Lines 160-162: please revise to make clear that this is a triple mutant

The requested modifications have been made in line 172.

5) Fig 3 & 5: The spot-titers represent one biological replicate. How many were performed with similar outcomes? Could these data be quantified to consider variability and potential errors in dilution. See for instance triple mutant + pSpyM69Q,M87Q, where the 10-fold dilution gives a full spot but the 100-fold dilution not a single colony.

The spotting experiments are representative of three independent replicates. This information was missing in the initial version of the manuscript and has now been added in the revised version.
We believe that quantifying this type of experiment is not appropriate, as it is more qualitative. As noted by the reviewer, inconsistencies can sometimes be observed between 10-fold serial dilutions. We do not have a definitive explanation for this phenomenon, which appears only on plates containing antibiotics. One possible hypothesis is the existence of a collective protection mechanism that depends on a threshold: above a certain cell density, resistance is observed, while below it, sensitivity prevails. A dedicated study would be needed to fully investigate and understand this observation.

Reviewer #3:

This study explores the response of Escherichia coli to chlorite-induced oxidative stress, focusing on Spy and its regulation by methionine sulfoxide reductase (MsrPQ). Proteomic analyses show that Spy is the most overproduced protein during chlorite stress. Spy expression is specifically induced by chlorite via the BaeRS stress-response pathway, distinguishing it from responses to other reactive species. The authors propose that chlorite oxidizes methionine residues in Spy, impairing its molecular chaperone activity, but this oxidation is reversible through MsrPQ. Mutational analyses suggest that while N-terminal methionines are non-essential, cradle methionines (Met69, Met87, Met108) are crucial for Spy's function. Methionine oxidation mimics (Met-to-Gln mutants) result in loss of function, phenocopying Spy-deficient strains. In vivo and in vitro assays also imply that oxidized Spy cannot stabilize substrates or prevent protein aggregation, suggesting the critical role of cradle methionines.

This study identifies a chlorite-specific stress response mechanism in E. coli, where MsrPQ may preserve Spy's function by reversing methionine oxidation. However, several issues must be addressed to substantiate the conclusions.

We thank the reviewer for the thorough analysis of our study. We have addressed the comments in the revised version of the manuscript, which we believe has significantly strengthened both the quality of the work and the robustness of our conclusions.

Major Issues:

1. The band shift observed in SDS-PAGE suggests Spy oxidation in the absence of MsrPQ, but this is insufficient. Methionine oxidation in Spy should be confirmed using peptide mapping analysis (LC-MS/MS).

This crucial point, also raised by the first two reviewers, was addressed using mass spectrometry analysis. Our results provide evidence of methionine oxidation in vivo. All of these results are presented in Figure 1E and described in lines 139-145.

2. While mutational analyses suggest cradle methionines are crucial, the study lacks data on the activity of oxidized Spy (e.g., in vitro anti-aggregation assays using H_2O_2-oxidized Spy with substrates like MDH and citrate synthase, avoiding reducing agents). Aggregation of these proteins can be induced through temperature shifts or urea dilution without DTT (e.g., Nat Struct Mol Biol. 2011, (3):262-9; J. Biol. Chem. 2020, 295(42) 14488-14500). Additionally, it is interesting to investigate whether MsrPQ can recover Spy activity in vitro.

We thank the reviewer for this suggestion, which we have implemented. Spy protein was oxidized *in vitro* using H_2O_2 to generate the oxidized form (Spy-ox), which was then tested for its chaperone activity. Since methionine sulfoxides (Met-O) cannot be reduced by DTT alone, but only by enzymes such as MsrA and MsrB (in the presence of DTT), or MsrP (in the presence of benzyl viologen and dithionite), the anti-aggregation assay using DTT is compatible with the use of Spy-ox and does not result in non-specific reduction of Met-O. Therefore, we did not need to set up alternative assays as originally suggested. Our results show that Spy-ox—whose Met-O status was confirmed by mass spectrometry—has lost its chaperone function. Interestingly, following repair by MsrP in the presence of a complete electron donor system (BV and dithionite), Spy regained its chaperone activity. This

experiment, which now constitutes a central part of our revised study, significantly strengthens our conclusions. All of these results are presented in Figure 4 C-D and described in lines 222-232.

3. The physiological significance of Spy in response to reactive chlorine species (RCS) remains unclear despite evidence of Spy induction and its role in antibiotic resistance in a Δskp ΔfkpA background. Further exploration and data of its specific roles under RCS stress is needed.

We agree with this valuable comment. However, in agreement with the editors of PLOS Biology, demonstrating the physiological significance of Spy overproduction under chlorite stress was not required for a Short Report.
That said, we acknowledge that the lack of a strong phenotype in the *spy* single mutant prevented us from pursuing a genetic complementation screen, as noted in our manuscript. Despite our efforts, we were unable to detect any chlorite sensitivity phenotype in the *spy* mutant. We suspect that functional redundancy with other periplasmic chaperones may be masking a potential phenotype. We plan to continue investigating the physiological relevance of Spy induction in future studies.

Specific Comments:
1. Title and Key Claims (Lines 138-139, 207-209): These statements are too definitive given the lack of direct evidence for methionine oxidation in Spy under RCS stress.

Thanks to the valuable suggestions made by the reviewers during the review process, we have included new results in this revised version that further support both our title and key messages. We agree with the reviewer that, in the original version, some of our conclusions may have appeared overly definitive. However, in light of the new evidence presented, we believe that maintaining the title and main conclusions is now well supported. We hope the reviewer will find these additions convincing and agree with our decision.

2. Lines 156-158: Previous studies (e.g., mBio. 2021, 12(5):e0213021) reported observable phenotypes upon spy deletion. Why were no such phenotypes detected or examined in this study?

The reviewer is correct: in the *mBio* paper by He et al. 2021, the *spy* mutant is reported to be more sensitive to novobiocin and polymyxin B. We were unable to reproduce such a strong phenotype under our experimental conditions, which prevented us from using these phenotypes in our study. That said, in He *et al.*, the Spy variants were tested using a multicopy suppression assay in a Δ*skp* Δ*fkpA* Δ*spy* triple mutant. We chose to follow the same strategy in our work, as the phenotypes observed in this background are sufficiently robust for use in multicopy suppression experiments.

Other phenotypes of the single *spy* mutant have also been reported, such as tannin sensitivity, described in the original study that led to the discovery of Spy (Quan et al. 2011). Although the tannin we used (Sigma #103040) clearly induces *spy* expression (see S1 Fig), we were again unable to detect a sufficiently strong sensitivity phenotype. This could be due to differences in the tannin origin or the culture medium used (LB vs. MOPS). Overall, we suspect that functional redundancy with other periplasmic chaperones may be masking the phenotypes associated with *spy* deletion.

3. Lines 158-160: Provide additional context to explain the rationale for using this method.

As explained above, the multicopy suppression assay performed in a Δ*skp* Δ*fkpA* Δ*spy* triple mutant is the most robust method reported in the literature for testing Spy variants. For this reason, we chose to use this approach. We hope that the revised version makes our methodological choice clearer. The changes can be found on lines 165-168.

4. Figures 1B, 1C, and 3: Clarify if SDS-PAGE was performed under reducing conditions. Was the Spy band shift reversed by DTT or purified MsrPQ? Include housekeeping proteins as loading controls (e.g., FtsZ for whole-cell lysates, MBP for periplasmic fractions).

We have clarified in this revised version that the SDS-PAGE analyses were performed under reducing conditions, in the presence of both DTT and β-mercaptoethanol. These changes can be found on lines 95, 384 and 604.
As mentioned earlier (see point 2), DTT alone does not reduce Met-O. In contrast, MsrP, together with an appropriate electrons donor system (BV/Dithio), is able to reverse the migration shift of Spy, as shown in the figure below. In the revised version, we have also included mass spectrometry data demonstrating the levels of Met-O after oxidation and subsequent repair by MsrP.
Regarding the loading control, we have added, across all immunoblots, the detection of a non-specific band recognized by the anti-Spy antibody on the same membrane. The use of the non-specific band as a loading control has been indicated in the figure legends.

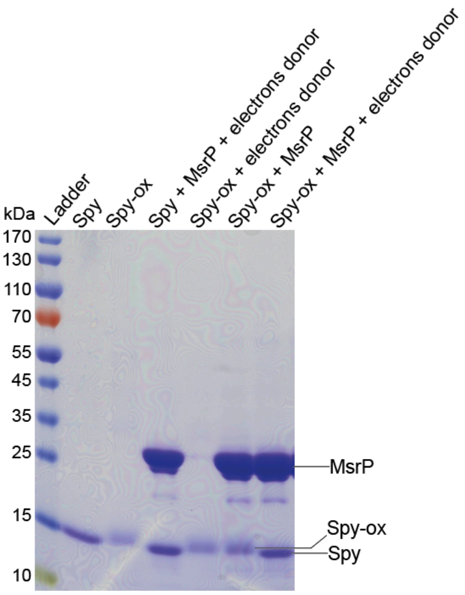

C.N.R.S.- L.C.B. 31 Chemin Joseph Aiguier, 13402 Marseille CEDEX 20, France.
Tel. : (33) 4.91.16.40.77. Fax : (33) 4.91.71.89.14
Email : lcb-sec@imm.cnrs.fr

5. Figures 5 and 6: Confirm and report whether wild-type and mutant Spy proteins are expressed at equivalent levels using immunoblotting.

We confirm that all Spy variants (M to A or M to Q) are produced at levels comparable to the wild-type protein. Only the Q100L variant appears to be less abundant. Immunoblots have been added for each experiment in which the variants were tested, which correspond to Figures 3D, 4B, and S2 Fig in the revised version of the manuscript. These immunoblots are shown below.

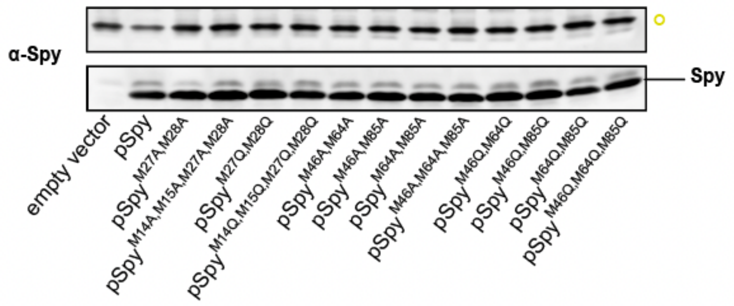

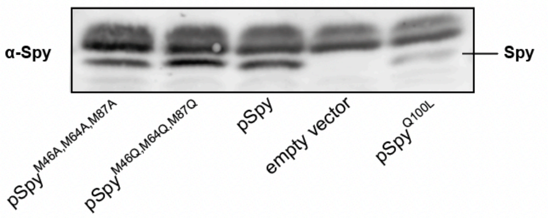

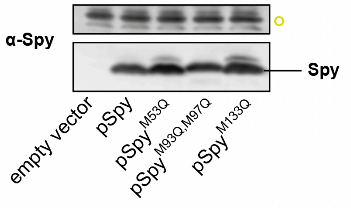

6. Line 32: Replace "reveal" with "suggest."
In light of the new results obtained during the revision, we have decided to retain the term *"reveal"*. We agreed with the reviewer that, in the initial version, this wording may have been too strong. However, we now believe that the new data support its use, and we hope the reviewer will agree with this choice.

7. Line 33: "Demonstrate" is too strong; consider revising.
For the same reasons outlined in point 6 above, we have decided to retain the word *"demonstrate"*.

8. Replace "chaperones" with "molecular chaperones".
We have added the word "molecular" before "chaperone" throughout the revised text, except in a few cases to lighten the wording, particularly in titles.

9. Lines 103, 105, 108: Correct figure references to Fig. 1C.
The requested modifications have been made.
10. Line 190: Specify "penicillin V."
The requested modifications have been made.

11. Lines 310, 327: Indicate if reducing agents (DTT, β-mercaptoethanol) were included in buffers.

The requested modifications have been made.

12. Lines 345-346: Correct to "0.2 M Tris-HCl, 1 M sucrose, 1 mM EDTA."

This paragraph has been removed in the revised version of the manuscript.

13. Lines 344-349: Provide details on immunoblotting for the periplasmic fraction.

The inclusion of the spheroplast preparation protocol in the initial version of the manuscript was an error. In fact, the proteomic analysis was performed on a whole-cell extract. Although experiments using periplasmic extracts were conducted during the course of the study, they are not presented in this manuscript. However, some of these results were shared during oral presentations at scientific conferences. As a result, the corresponding paragraph has been removed.

C.N.R.S.- L.C.B. 31 Chemin Joseph Aiguier, 13402 Marseille CEDEX 20, France.
Tel. : (33) 4.91.16.40.77. Fax : (33) 4.91.71.89.14
Email : lcb-sec@imm.cnrs.fr

Reviewer #4:

This short report contains a straightforward set of simple but carefully done experiments that demonstrate that the periplasmic chaperone Spy is both induced by chlorite stress and is susceptible to methionine oxidation by chlorite. As chlorite damages many proteins leading to their inactivation and aggregation it seems reasonable that a chaperone would be induced by a protein damaging agent and that since the Spy chaperone itself is a protein that it is inactivated by chlorate seems reasonable. These results should spur future research. For instance the observation that Spy is cross linked by chlorate is intriguing as Spy does not contain any cysteine residues and I am unaware of methionine modification in a way that would crosslink proteins. This result should be highlighted more in the paper, do their mass spec experiments reveal any cross linked peptides?

We are pleased to see that the reviewer considers our work to be well conducted and that the results are likely to encourage future research. We share the reviewer's interest in the presence of a Spy dimer observed under chlorite stress, despite the use of a denaturing gel.
As specialists in methionine oxidation, this is the first time we have observed such dimerization, suggesting that methionine sulfoxide is likely not involved in the crosslinking. Despite numerous mass spectrometry analyses, we have not yet been able to determine the molecular basis of this phenomenon.
As additional information, we would like to share in this response a result that is not included in the manuscript. In this experiment, we show that the use of a constitutively active *baeS* mutant, which leads to Spy overproduction, does not result in dimer formation. However, the addition of chlorite clearly induces dimerization. This strongly suggests that chlorite is directly responsible for the crosslinking.
We will continue our efforts to elucidate the molecular mechanisms underlying this intriguing observation.

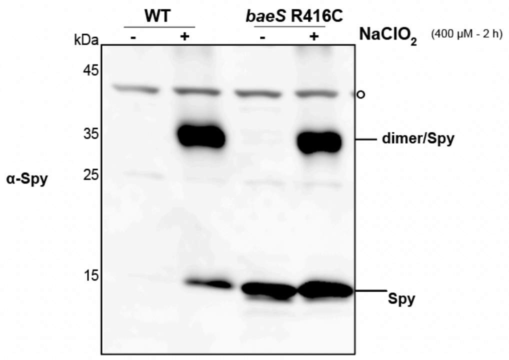

C.N.R.S.- L.C.B. 31 Chemin Joseph Aiguier, 13402 Marseille CEDEX 20, France.
Tel. : (33) 4.91.16.40.77. Fax : (33) 4.91.71.89.14
Email : lcb-sec@imm.cnrs.fr

I have a few suggestions for improvements:
The precise basis of the phenotypic screen should be made more clear for the naive reader.

To assist non-specialist readers, we have revised the the schematic representation of the genetic screen to make it, we hope, even clearer and easier to understand.

Supplementary table 1 needs a lot of work to make it user friendly. For protein validation they required "At least 2 peptides (best hit) and at least one being specific" what exactly does this mean?
What are the # peptide, # specific peptide and # total peptide columns exactly, they don't seem to add up.
The color scheme is not at all clear, which proteins are in which color classes? Many of the column headings are not immediately clear. Because of these issues It is difficult to determine what the unlabeled proteins in the upper right portion of the figure 1A are, ie those that are induced strongly in fold change and that have significantly p values. Spy though strongly induced in fold is barely above the threshold p value line. It would be good to label about 10 more proteins in this quadrant and in general make supplementary table 1 much more user friendly.
An indication of the fold overproduction of Spy protein by the plasmid overproducer should be stated.

We proposed a new simplify version of the supplementary table 1 with a legend. "A protein is validated when at least two peptides were found in one of the sample and at least one of these peptides is unambigously assigned to this protein (this peptide is then proteotypic of the protein)."
In Figure 1A, we highlighted the most overproduced and underproduced proteins in red and blue, respectively. Additionally, we identified the top 10 proteins in each category (red and blue) to provide clearer and more direct information.

We hope that these changes will meet the reviewer's expectations and lead to a better understanding of the proteomics results.

In the discussion, I think that postulating the N terminal methionine are oxidant sponges is one possibility but a simpler interpretation is that as non-conserved residues they are simply not important for Spy function.

We have made changes to the Discussion section to introduce this possibility. Accordingly, we have added the following sentence: "*Another possibility, not mutually exclusive with the previous one, is that these non-conserved methionine residues are simply not essential for Spy function.*"

One source of chlorate and chlorite is anthropogenic but the related hypochlorous acid is an key component of the host immunes system of mammals as neutrophils release high concentrations of hypochlorous acid when microbial invasion occurs, this biological source should be mentioned.

We agree with the reviewer regarding the production of RCS, including hypochlorous acid by neutrophils. However, as shown in Figure S1A, HOCl does not induce *spy* expression. For this reason, we prefer not to include this possibility in the manuscript.

C.N.R.S.- L.C.B. 31 Chemin Joseph Aiguier, 13402 Marseille CEDEX 20, France.
Tel. : (33) 4.91.16.40.77. Fax : (33) 4.91.71.89.14
Email : lcb-sec@imm.cnrs.fr
