# Supplementary material for: Repair of oxidized methionine residues in the chaperone Spy maintains periplasmic proteostasis under chlorite stress in Escherichia coli
Source: PLoS Biol. 2025 Sep 29;23(9):e3003411. doi: 10.1371/journal.pbio.3003411 (PMC12503329; doi:10.1371/journal.pbio.3003411)
Supplement: Attachment — Submitted filename: Response to Reviewers REV2.pdf [file pbio.3003411.s013.pdf]

Responses to editor and reviewers (comments in red / responses in black), modifications in the text are highlighted in yellow.

Referee #1

The authors have made a commendable effort to address my previous concerns by providing additional experimental evidence. The conclusions are now significantly strengthened. In principle, I believe the manuscript is acceptable for publication. However, a few minor points should still be addressed:

We thank the referee for their assistance in the review process, for pointing out remaining typographical errors in the Met residue numbering, and for their suggestions to improve clarity in the text, which enhance the understanding of our results for a broad audience.

1. In lines 213, 218, 220, and 221, the mutant "M46Q/M68Q/M64Q" appears to be a typographical error. Shouldn't the correct mutant be "M46Q/M64Q/M85Q"? Additionally, in Figure 4B, M85Q is mistakenly labeled as M87Q in the western blot and should be corrected.

The corrections have been made in lines 216–228; the residues are indeed M46, M64, and M85.

2. I appreciate the inclusion of mass spectrometry data on the oxidation status of methionine residues in Spy. However, I am still curious about the nature of the identified modifications, whether they are methionine sulfoxide or methionine sulfone. It would be helpful to clarify the nature of the oxidation products. Currently, "methionine sulfoxide" is only mentioned in the abstract. It would improve clarity to define the specific oxidation type in the results section as well.

Indeed, we were not sufficiently clear about the nature of methionine oxidation. It concerns methionine sulfoxide; to our knowledge, methionine sulfone has never been reported in a protein under in vivo stress. The formation of sulfone in vitro requires very specific pH conditions. In this revised version, we have followed this recommendation, specifying the appearance of sulfoxide (+16 Da) in lines 132, 143, and 232.

3. For Figure 4, please provide a reference for the "complete electron donor system" and briefly explain its components or function, as this term may not be familiar to all readers.

We have added in the main text (line 238) the composition of the electron donor system used for MsrP recycling, and included the reference to the study characterizing MsrP enzymatic activity (Gennaris et al., doi:10.1038/nature15764).

4. Also in Figure 4, H_2O_2 was used to induce methionine oxidation. A brief explanation of why H_2O_2 was chosen instead of chlorite would be helpful for readers. Is it due to greater controllability or a more established methodology? Does it produce a comparable oxidation outcome to chlorite?

We have added details in the main text (line 230) regarding the use of H_2O_2. This is a well-established protocol that considerably reduces in vitro oxidation of other types of residues that could occur with chlorite or other oxidants.

C.N.R.S.- L.C.B. 31 Chemin Joseph Aiguier, 13402 Marseille CEDEX 20, France.
Tel. : (33) 4.91.16.40.77. Fax : (33) 4.91.71.89.14
Email : lcb-sec@imm.cnrs.fr

Aix Marseille Université
LABORATOIRE DE CHIMIE BACTÉRIENNE
cnrs

Reviewer #2:
The authors have responded well to all feedback. I recommend acceptance and applaud this beautiful study.

Thanks for the positive comments.

Reviewer #3:

The authors have addressed most of my initial comments; however, several important issues remain and should be considered for further revision:

1. The authors have added new MS-based data quantifying methionine oxidation at each residue in the wild-type and ΔmsrP strains (Fig. 1E). While these results confirm both site-specific Met oxidation and the antioxidative function of MsrP, the effect of chlorite stress on Met oxidation remains unclear, as no data are presented for the non-stress condition. If detecting Met oxidation in Spy Met residues is technically challenging in the absence of stress, a baeS R416C mutant strain—which constitutively activates the Bae pathway—could be a valuable tool to address this point.

The use of the *baeS* R416C mutant, which constitutively expresses *spy* to analyze methionine oxidation levels in Spy under no-stress conditions, is indeed a very smart idea. Following the recommendation of the PLOS Biology editors, we did not conduct this experiment; nevertheless, we will consider this approach in future studies.
In Figure 4D, we do analyze the basal level of Spy oxidation in the absence of stress, after plasmid-driven overproduction and protein purification. The oxidation level ranges from 25% to 50% of Met-O depending on the residue positions. While this level may be slightly overestimated due to non-physiological oxidation during overproduction and purification, it provides a general idea of the oxidation rate.

2. In the immunoblots of Spy variants, it is unclear whether monomers or dimers are being shown, as molecular weight markers are not indicated. Additionally, the results in Fig. 4B appear inconsistent with those in Figs. 3D and S2. Fig. 4B also lacks a loading control. The use of non-specific protein bands as loading controls is questionable, as their expression may vary under different culture conditions or genetic backgrounds. For example, in the second lane from the left in Fig. 3D, the intensity of the non-specific band is noticeably reduced compared to the others, suggesting either a biological effect or a loading inconsistency. If antibodies against housekeeping proteins are not available, CBB staining of the SDS-PAGE gel can serve as an alternative method to confirm equal protein loading.

We thank the reviewer for this comment. We have added molecular weight markers in Figures 3D, 4B, and S3 (formerly S2). These correspond to Spy monomers, which is consistent with our response to the comments on the first version, showing that Spy dimers appear only under chlorite stress. In these figures, Spy production was plasmid-driven and performed in the absence of chlorite.

Figure 4B shows Spy production from the pCDFT plasmid in an HW38 strain (ΔhsdR ΔampC Δspy), which was provided by Shu Quan's laboratory in consultation with James Bardwell's group. In contrast, Figures 3D and S3 (formerly S2) result from Spy production from the pTrc99A plasmid in an MG1655 Δskp ΔfkpA Δspy strain. These differences in production systems may explain the variations in band patterns between Figure 4B and Figures 3D and S3.

C.N.R.S.- L.C.B. 31 Chemin Joseph Aiguier, 13402 Marseille CEDEX 20, France.
Tel. : (33) 4.91.16.40.77. Fax : (33) 4.91.71.89.14
Email : lcb-sec@imm.cnrs.fr

In this revised version, we have included the full membranes of these blots (in S2, S3, and S5), allowing visualization of the pattern differences detected by the anti-Spy antibody depending on the experiment. Furthermore, we noted that, depending on the commercial batch of the anti-Spy antibody (Clinisciences), there was variability in the detection of non-specific bands.
The loading control has been added to Fig 4B.
Additionally, in agreement with the journal editors, we have included the full blots probed with the anti-Spy antibody, which show numerous non-specific bands and thus help to assess potential loading issues. We have also moderated, where necessary, the comparisons of loading between lanes in the text (lines 183–185; 191–193; 218–221).

3. The responses to my original comments #12 and #13 are not satisfactory. The authors state that periplasmic fractions were used for the immunoblots (Lines 90 and 602), but this does not fully address the concern raised.

The mention of using the periplasmic fraction for the immunoblot in the text was a deletion oversight between the original version and R1. We thank the reviewer for pointing this out, and we have corrected this error in the current R2 version. The immunoblots were indeed performed using total extracts, as confirmed by the presence of MsrP precursor visible in the raw image (S1_raw_images.pdf) of the blot probed with the anti-MsrP antibody in Figure 1B.

4. Lines 227-229 should be revised for accuracy. Although Spy-ox showed reduced activity, it still exhibited clear suppression of aggregation (Fig. 4C). The current description does not adequately reflect the data.

The requested modifications have been made in lines 235-237.

C.N.R.S.- L.C.B. 31 Chemin Joseph Aiguier, 13402 Marseille CEDEX 20, France.
Tel. : (33) 4.91.16.40.77. Fax : (33) 4.91.71.89.14
Email : lcb-sec@imm.cnrs.fr
